# Sjogren’s syndrome in clinical trials of traditional Chinese medicine: protocol for the development of a core outcome set

**DOI:** 10.1186/s13063-021-05187-8

**Published:** 2021-03-26

**Authors:** Ming Liu, Ya Gao, Yuan Yuan, Shuzhen Shi, Kelu Yang, Jiyuan Shi, Jiarui Wu, Junhua Zhang, Jinhui Tian

**Affiliations:** 1 Evidence-Based Medicine Center, School of Basic Medical Sciences, Lanzhou University, No.199, Donggang West Road, Chengguan District, Lanzhou City, Gansu Province China; 2 Key Laboratory of Evidence-Based Medicine and Knowledge Translation of Gansu Province, Lanzhou, China; 3 Gansu University of Chinese Medicine, Lanzhou, China; 4 Evidence-Based Nursing Center, School of Nursing, Lanzhou University, Lanzhou, China; 5 Department of Clinical Chinese Pharmacy, School of Chinese Materia Medica, Beijing University of Chinese Medicine, Beijing, China; 6 Evidence-Based Medicine Center, Tianjin University of Traditional Chinese Medicine, No.312 Anshanxi Street, Nankai District, Tianjin City, China

**Keywords:** Sjogren’s syndrome, Traditional Chinese medicine, Protocol, Core set comes

## Abstract

**Background:**

Sjogren’s syndrome (SS) is a chronic autoimmune rheumatic disease with an incidence of 0.03 to 0.3%. In recent years, there are an increasing number of randomized controlled trials of traditional Chinese medicine (TCM) for SS. However, there are generally some problems in these published clinical trials: lack of reporting primary or long-term outcomes and the heterogeneous in different clinical trials’ outcome. Our study aims to determine the priority outcomes and standard TCM syndromes for all stakeholders and reach agreement on the COS and syndromes to be measured and reported in all future TCM trials in patients with SS.

**Methods and analysis:**

A phase-wise refinement approach will be used, consisting of three phases, yet complementary, sub-work phases, whereby each phase will inform the next coming phases. The following are the three phases: (I-a) identifying of a long initial list of outcomes through systematic literature review and semi-structured qualitative interviews and (I-b) identifying an initial list of TCM syndromes through (1) systematic literature review, (2) referencing ancient Chinese medical books, and (3) retrospective studies of medical records; (II) prioritization of outcomes using Delphi survey with different stakeholders, such as health professionals and patients; and (III) through consensus meetings with patients and professionals to agree on the final COS and TCM syndromes.

**Discussion:**

We summarized the actions of COS into three points: direct action, indirect action, and final action. After the final COSs is completed, we will publish this research in a journal to promote communication.

**Trial registration:**

Core Outcome Measures in Effectiveness Trials Initiative (COMET) number 1429. Registered on 01 December 2019.

**Supplementary Information:**

The online version contains supplementary material available at 10.1186/s13063-021-05187-8.

## Strengths and limitations of this study

1. The study seeks to determine the priority core outcome sets and standard TCM syndromes for all stakeholders.
2. In the process, we plan to use semi-structured qualitative interviews and Delphi survey.
3. We will consult ancient Chinese medical books for its relevant symptoms according to the most classic symptoms and signs of SS.
4. We will focus on core outcomes identified as important by former patients, as well as clinicians and researchers.
5. Patients will be only recruited at China.

## Introduction

Sjogren’s syndrome (SS) is a chronic autoimmune rheumatic disease, which is characterized by lymphocytic infiltration of exocrine glands as well as other organs that are association with the production of various autoantibodies in the blood [1]. The disease develops invisibly for months to years in most cases. The most common clinical manifestations are dry mouth, dry eyes, and swelling of the major salivary glands. Moreover, some patients manifest with signs and symptoms suggestive of liver, lung, and other organ damage. Multiple studies published in recent years show that SS, like other immune diseases, can severely affect a patients’ quality of life [2]. What is more, SS patients have a high rate of severe depression and anxiety [3]. In 2008, an epidemiological study estimated a prevalence of SS ranging between 0.4 and 3.1 million individuals in the USA [4]. A 2015 study showed that the overall prevalence of SS is 0.03 to 0.3% globally [5]. The serious injuries and rising incidence of SS have placed a huge burden on patients, families, society, and the world.

There is currently no cure or remitted agent for SS that exists. Therefore, the primary goals of therapy are limited to palliation of symptoms, prevention of complications, treatment of extra-glandular manifestations, and use of immunosuppressive therapy for patients based on the activity and severity of disease [6]. Traditional Chinese medicine (TCM), which is based on more than 2000 years of accumulated knowledge and practice, is becoming increasingly popular outside China [7], because TCM has unique advantages in the treatment of certain diseases. Recently, the 2015 Nobel Prize in Physiology or Medicine was awarded to Tu Youyou’s discovery and development of artemisinin [8]. This is a clear example and reminder of the significant potential that TCM holds. With this being said, the TCM approach, in relation to both diagnosis and treatment, fundamentally differs from western methods, and its effectiveness is difficult to validate under modern scientific methods [9]. For example, syndrome (Table 1) differentiation is the basis of developing therapeutic principles in TCM. Generally in TCM, there are some problems that often are faced during clinical trials: lack of reporting primary or long-term outcomes and heterogeneous outcomes in different clinical trials [10]. These challenges can lead to a series of undesirable consequences: (1) a limited ability to adequately compare and interpret clinical trial results, (2) hampered data pooling and subsequent unreliable meta-analysis, and (3) increased risk of selective outcome reporting as well as other issues [11, 12]. This hinders progress towards improvements in medicine and results in a waste of resources. As to best inform the evidence base, outcomes must be selected, defined, and measured consistently across studies of similar interventions in similar populations.

**Table 1:** The definition of Traditional Chinese Medicine syndromes

*What are TCM syndromes?*	
“Syndrome” is an abstract generalization of the pathological changes of a disease at a certain phase, which can reveal the essence of the disease in a deeper and more comprehensive way, and is the basis of clinical treatment of TCM. In clinical practice of TCM, syndrome elements are of great significance for diseases diagnosis and treatment, owing to different syndrome groups have different metabolic characteristics and susceptibility to diseases.	
*Example*	Blood stasis syndrome
	Deficiency of Qi and Yin syndrome
	Qi deficiency syndrome

Core outcome sets (COSs) refer to the minimum results that should be measured and reported in clinical trials in a specific area of healthcare [13]. At present, COSs are increasingly being developed to identify outcomes important to decision makers, improve outcome reporting, and standardize definitions and measures [14]. In 2010, the Core Outcome Measures in Effectiveness Trials (COMET) Initiative was founded and it committed to the development, implementation, dissemination, and update of COSs by published the consensus-based standards for the selection of health measurement instruments (COSMIN) [15]. The “COMET Handbook: version 1.0” was developed by the COMET Initiative in 2016 [16]. The number of completed COSs is rapidly increasing and covers the field of rheumatism, cancer, pain, trauma, etc. [17, 18].

In the face of the current dilemma, the development of COSs can solve the problem of clinical trials of TCM. Coincidentally, in 2017, Consolidated Standards of Reporting Trials for Chinese Herbal Medicine Formulas (Revised) recommended detailed reporting of TCM syndrome outcomes [19]. In our study, we aim to develop a COS as well as standard TCM syndromes for SS.

## Aim and scope

The study seeks to determine the priority outcomes and standard TCM syndromes for all stakeholders. It also aims to reach agreement on the standardized COS and syndromes that are needed to be measured and reported in all future TCM trials for patients with SS.

We developed the scope of the COS through the criteria recommended by COMET [16]. The scope of the COS-TCM is as follows: (i) health condition: SS; (ii) population: patients (age ≥ 18 years) with SS; (iii) types of interventions: therapies based on the therapeutic principles of TCM, including traditional herbal medicine, acupuncture, cupping, Qigong, and other non-drug therapies; and (iv) setting: randomized controlled trials (RCTs).

## Method and analysis

The content of this protocol follows the Core Outcome Set Standards for Protocol Items (the COS-STAP Statement) in the protocol [20]. This study has been registered on the COMET database, with the registration number 1429 (http://www.comet-initiative.org/studies/details/1429). If protocol amendments occur, the dates, changes, and rationales will be tracked in the COMET database.

### Steering group

A steering group, including five experts (two TCM experts, a Western medicine expert, a methodologist and two policy makers), will combine efforts to guide the development of the COS. Their main job is to review and confirm the research protocol, make decisions if confusion or disagreements arise, and attend consensus meetings.

### Patient and public involvement

We will recruit patients with SS to participate in semi-structured interviews, Delphi survey, and consensus meetings.

### Design

**Fig. 1:**
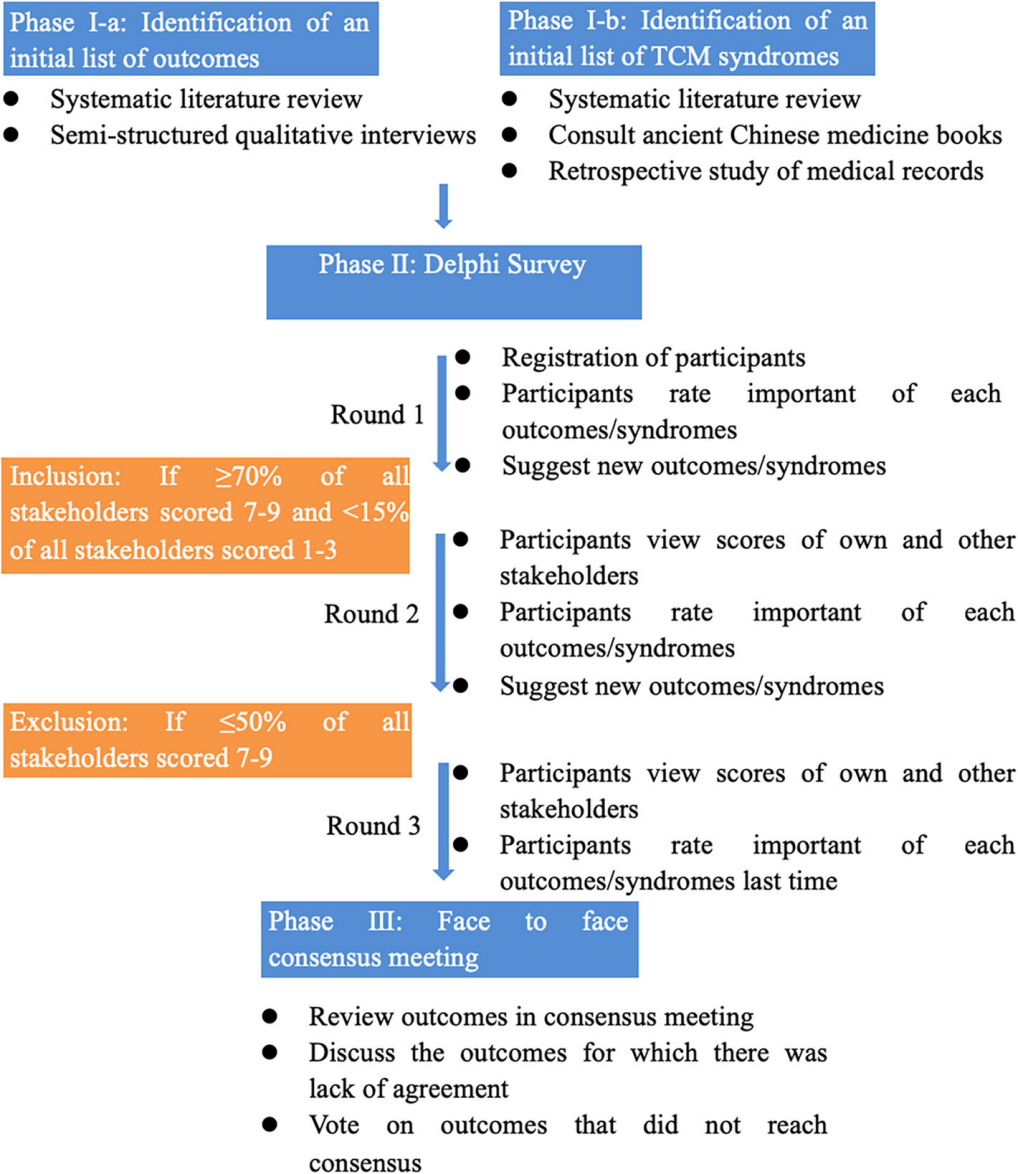
Key phases in process

A phase-wise refinement approach will be used, consisting of three phases, yet complementary, sub-work phases, whereby each phase will inform the next coming phases. The flowchart is shown in Fig. 1. The following are the three phases:

- (I-a) Identifying of a long initial list of outcomes through (1) systematic literature review and (2) semi-structured qualitative interviews.
- (I-b) Identifying an initial list of TCM syndromes through (1) systematic literature review, (2) referencing ancient Chinese medical books, and (3) retrospective studies of medical records.
- (II) Prioritization of outcomes using Delphi survey with different stakeholders, such as health professionals and patients.
- (III) Through consensus meetings with patients and professionals to agree on the final COS and TCM syndromes.

#### Phases I-a: Identifying an initial list of outcomes

We plan to identify the initial list of outcomes through two steps: (1) a systematic review and (2) a semi structured interview.

##### Step 1: Systematic review

*Inclusion criteria and exclusion criteria*

The inclusion and exclusion criteria for the literatures are shown in Table 2.

**Table 2:** The inclusion and exclusion criteria of systematic review

Items	Inclusion criteria	Exclusion criteria
P	Patients with Sjogren’s syndrome [21, 22] (age ≥ 18 years)	Other patients
I	Any sort of TCM*	Exercise treatments such as walking and meditation
C	Any sort of intervention or placebo (or nothing at all)	None
O	Outcome measurement time or outcome definition instruments can be extracted	The main purpose of the study is to assess the mechanism or pharmacokinetics of the drug
St	Randomized controlled trials	Full-text cannot be obtained
L	Published in Chinese or English	Published by other language
Ss	Sample size ≥30 in each group	Sample size of one group is less than 30

*P*, patients; *I*, intervention; *C*, comparison; *O*, outcome; *St*, study types; *L*, language; *Ss*, sample size; *TCM*, traditional Chinese medicine

*The intervention of traditional Chinese medicine only included herbal drug, herbal patch, acupuncture, Tuina, and Chinese patent drug

*Search strategy*

A study showed that literature searches could be improved by searching more sources [23]. Hence, we searched through four English and three Chinese databases: PubMed, Embase, Cochrane Library, Web of Science, China Online Journals, China Academic Journals Full-text Database, and China Biomedical Literature Database. In addition, we will search the China Clinical Trial Registration Center. The retrieval time range is from inception until the date that the searches were conducted. The search strategy of PubMed as an example is shown in the Additional file 1.

*Study selection*

Due to the absence of a study design filter, a large number of irrelevant records could be identified by our search strategy. Based on the predetermined review inclusion criteria, the titles and abstracts of all the retrieved records will be screened through the EndNote X8 literature management software (Thomson Reuters [Scientific] LLC, Philadelphia, PA). Studies that still cannot be judged will be identified by reading the full text. All manuscripts will be checked independently by two reviewers. Discussions will be used to resolve disagreements between two reviewers or by consulting a third reviewer.

*Data extraction*

The data will be extracted independently by two reviewers. The following information will be extracted from each included study: the first author’s name, publication time, sample size, interventions, outcomes (if possible, the primary and secondary outcomes will be identified, separately), the definition of outcomes, outcome measurement instruments, measurement time (intervention duration and follow-up time), and TCM syndromes (if provided). Discussions will be used to resolve disagreements between two reviewers or through adjudication of a third reviewer. If the data is incomplete, a reviewer will contact the corresponding author to obtain the complete data via email.

*Data analysis and presentation*

After the data extraction is completed, the outcomes will be standardized and classified into different domains. Discussions will be used to resolve disagreements between two reviewers or through advisory Steering Group. See Table 3 for the specific methods.

**Table 3:** Data analysis methods and steps

Steps	Specific method
1	Translation of English ending indicators into standard Chinese names. (If no relevant terminology, the steering group will be consulted.)
2	Separate composite outcomes will be divided into a single outcome.
3	Remove outcomes that define repetition.
4	Two authors will group the outcomes together into appropriate outcome domains
5	The frequency of each individual outcome and the number of outcomes belonging to an outcome domain will be assessed.
6	The outcome domains and their outcomes will be reviewed by the Steering group.

##### Step 2: Semi-structured interviews

Similar to the development of the guideline, it is important to acquire the patient’s opinions regarding the development of COSs [24]. Therefore, we will invite the patients with SS to attend a semi-structured interview, to obtain their opinions of the outcomes of SS that should be measured in clinical trials.

*Participant selections*

To ensure that the patients are diagnosed correctly, we will select and interview patients that have been admitted in the rheumatism and immunology department pertaining to a tertiary hospital. We will also interview patients aged 18 or above who have had previous TCM treatment. Patients must have more than 3 years of medical history. Patients will be excluded if they are reported to have (1) a serious disease, which prohibits participation in any clinical trial; (2) a serious mental illness, psychosis, or a communication disability; and finally (3) if the patient refuses to participate.

*Sampling and recruitment*

Each interviewee will fill out a brief questionnaire to record their demographic characteristics and that they consent to take part in the semi-structured interview. First, we will randomly select 30 eligible patients as interviewees and obtain their consent and signature to accept face-to-face or telephone interviews. A series of interviews will be conducted until no new results are found in three consecutive interviews, which is called “data saturation” [25]. At this time, the number of interviewees who have participated in the interview is the final number. If the initial number of 30 interviewers is not enough, we will continue to randomly select in the eligible patients to participate this semi-structured interview until the data is saturated.

*Outline of the semi structured interviews*

The outline of the semi-structured interviews is shown in Table 4.

**Table 4:** The outline of the semi structured interviews

Numbers	Questions
1	When were you diagnosed with Sjogren’s syndrome?
2	What inconveniences have you experienced after being diagnosed with Sjogren’s syndrome?
3	What kind of treatment have you received?
4	What is your expected treatment effect?
5	What do you think is the biggest side effect of your current treatments?
6	What is the most important outcome for you?

*Data collection and analysis*

All of interviews will be tape recorded and transcribed verbatim. Data collection will be undertaken concurrently with data analysis through thematic analysis. Firstly, two reviewers will independently review the transcripts line by line and code the words and phrases related to important outcomes or treatments. Secondly, narrative explanations of the effects of SS and treatments on the patients’ lives will be interpreted by the process of constant comparison to identify outcomes that are important to patients. Thirdly, two reviewers will identify whether these outcomes are new. Any disagreements between the two reviewers will be resolved by discussion or with the help of an advisory steering Group. If there are differences to any systematic reviews, they will be added to the initial list of outcomes.

#### Phases I-b: Identifying an initial list of traditional Chinese medicine syndromes

There are three steps to identifying an initial list of TCM syndromes, which include reviewing a systematic review, consultation with Ancient Chinese Medical Books, and review of retrospective studies of medical records.

##### Step 1: Systematic review

*Inclusion criteria and exclusion criteria*

The inclusion criteria and exclusion criteria are similar to what was described in Phase I-a.

*Search strategy*

The search strategies are the same as clarified in phase I-a.

*Data extraction*

In addition to extract the names of TCM syndromes, we will extract the syndrome diagnostic criteria, syndrome evaluation items (such as symptoms, tongue coating and pulse), and syndrome measurement methods. Any disagreements between the two reviewers will be dealt with by means of discussion or through an advisory Steering Group.

*Data analysis and presentation*

Similar to the process of analyzing outcomes from systematic reviews, we will refer to “Traditional Chinese Internal Medicine” to standardize the syndrome. In addition, we will supplement syndromes from several current TCM books, such as “Guidelines for Clinical Research of New Drugs in Traditional Chinese Medicine” and “Traditional Chinese Medicine Syndromes.”

##### Step 2: Consult ancient Chinese medicine books

SS did not have an accurate disease name in ancient China. We will consult ancient Chinese medical books for its relevant symptoms according to the most classic symptoms and signs of SS. We will select the types of ancient Chinese medical books that require to be reviewed in two ways: (1) refer to “Chinese Ancient Traditional Chinese Medicine Books” (Including: Treatise on Febrile and Miscellaneous Diseases, Syndrome of traditional Chinese Medicine) and (2) consult TCM experts. Two reviewers will independently complete this work. Discussions will be used to resolve disagreements between two reviewers or by means of an advisory Steering Group. If there are differences to the systematic review, they will be added to the initial list of symptoms.

##### Step 3: Retrospective study of medical records

We will conduct a retrospective study of medical records in the last 3 years in two tertiary Chinese medicine hospitals. The purpose of this study is to obtain the names of TCM syndromes of SS from the perspective of the clinician. There are no other restrictions if all cases that were originally diagnosed as SS meet the requirements. If there are two or more syndromes, only the first syndrome is extracted; two reviewers will independently complete this work. Discussions will be used to resolve disagreements between two reviewers or by means of an advisory Steering Group. If there are not enough medical records in 2 hospitals for 3 years, we will expand the year, for example 5 or 10 years. If there are not enough cases for these 2 hospitals, we will join other hospitals. If there are differences from this part to the process of systematic review, they will be added to the initial list of symptoms.

#### Phases II: Delphi survey

The Delphi survey is an iterative consensus technology. The survey consists of several successive rounds of questionnaires, which will be answered by a panel of participants with relevant expertise [26]. It has two advantages when used in the development of COSs: (i) it can avoid situations where a select few people lead the discussion or other individuals feel obligated to agree with the opinions of senior members and (ii) it can facilitate wide international participation [27]. In our study, the questionnaire will be completed using the Delphi Manager: a web-based system designed to facilitate the building and management of Delphi surveys.

##### Stakeholder selection

Representatives of four particular stakeholder groups will be invited to participate in the Delphi survey: patients (Medical history of more than 3 years); clinicians have more than 10 years of work experience in tertiary hospitals (TCM clinicians, western medicine clinicians and integrative medicine clinicians); nurse have more than 5 years of work experience in tertiary hospitals; researchers with a master’s degree or above who have at least one peer-reviewed publication on the topic of SS (Whether TCM or Western Medicine or Integrative Medicine). There is no restriction on the geographical area of participants.

##### Sampling strategy

To our knowledge, there is currently no standard sample size calculation method in the Delphi survey [16]. Referring to the previous COSs, we plan to select 50 stakeholders for each stakeholder group. Because other stakeholders find it challenging to understand TCM terminology, only clinicians and researchers (worked in TCM and integrative medicine) will be invited to respond to the questionnaire of core TCM syndrome outcomes.

##### Consensus standards

We define the consensus standards according to previous related research. If 70% or more of the participants scored outcomes as 7–9, and < 15% of the participants scored the outcomes as 1–3, the COSs or TCM syndromes will be included. If 50% or less of the participants scored the outcome as 7–9, the COS or TCM syndromes will be excluded. Anything else can be used as the outcome of updating COSs in future.

##### Delphi study round 1

We will develop questionnaires for the list of candidate outcomes and TCM syndromes, separately. The participants are same, and only some answer the section on TCM syndromes, and some answer the section on COS. Each outcome or syndrome will have an associated plain Chinese definition. We also will collect participants’ demographic information in the round 1 questionnaires. Firstly, participants who agree will be invited to register in the Delphi Manager; then, they will receive an email linking to an online survey. The participants will be asked to score all of the items according to the Likert scale [28]. The scores 1 to 3 represents “not important for inclusion in the COSs,” 4 to 6 represents “important but not critical for inclusion in the COSs,” and 7 to 9 represents “critical for inclusion in the COSs”. An “unable to score” category will also be included, which allow an option for participants who may feel that they do not have the level of expertise to score certain outcomes. In addition, a participant is allowed to add two additional outcomes or syndromes that are not included in the list of candidate outcomes and TCM syndromes.

In regard to the period of the round 1 Delphi survey, we plan it to last for 3 weeks. At the end of second week, we will remind the participants to complete the survey by sending them an email or message. However, if the response rate is < 80% at the end of third week, we will keep the Delphi round open for a longer period of time to reduce attrition bias.

##### Analysis of round 1

The round 1 of responses will be collected and summarized using descriptive statistics. The distribution of scores for each outcome will be calculated separately for the whole Delphi survey and each stakeholder group. If more than 10% of participants from any stakeholder group, who considered the outcomes or symptoms to be important (scored ≥4), will be included in the second round of the Delphi survey. Any other outcomes or syndromes presented by the participants will be reviewed and discussed by the Steering group and research team and will be collectively included in the second round.

##### Delphi study round 2

Only participants who have completed round 1 will be invited to participate in the second round. These participants will again be contacted by email with a link to the online survey. Before scoring, participants will be given their previous rounds’ score and a histogram summary of the responses for each group and all groups combined. Then, they will ask to score the outcomes and syndromes from round 1. The remaining processes of the second round are the same as the first round of Delphi survey.

##### Analysis of round 2

Like the analysis of round 1, the distribution of scores for each outcome will be calculated separately for the entire Delphi survey and each stakeholder group. Outcomes and syndromes that meet the consensus standards will be directly used as the content of the consensus meeting. The remaining outcomes and syndromes will enter the round 3 of the Delphi survey. The participants of round 3 will again be contingent upon completing the survey in round 2.

##### Delphi study round 3

Participants will be contacted again via email with links to online survey. For each outcomes and syndromes, they will once again see a histogram summary of the responses for each group and for all groups combined from the rounds 2, as well as a reminder of their round 2 score. At this point, the participants will be asked to rescore each outcomes and syndromes, again from 1 to 9. Participants will be given 3 weeks to complete the round 3 process. At the end of the second week, we will remind participants to complete the survey by sending them an email or SMS message. However, if the response rate is < 80% at the end of third week, we will keep the Delphi rounds open for a longer period of time (at least 2 weeks) to reduce attrition bias.

##### Analysis of round 3

The analysis process follows the same process as the previous two rounds. Outcomes and syndromes will be classified as consensus in, consensus out, or no consensus. Combining the consensus results of the previous two rounds, the distribution of scores and consensus result for each outcomes and syndromes will be displayed by group and overall and used to structure the final consensus meeting.

#### Phases III: Consensus meeting

A face-to-face consensus meeting with key stakeholders will be held after completion of the Delphi process. The meeting will be chaired by an independent chair to finalize the COS and the TCM syndromes.

##### Participants

A purposive sample of approximately 15 stakeholders will be included: (i) clinicians have more than 15 years of work experience in tertiary hospitals (one TCM clinicians, one western medicine clinicians, one integrative medicine clinicians); (ii) patients (at least three) who completed three rounds of Delphi surveys; (iii) policy-makers and service providers involved in formulating policies/providing services related to TCM; (iv) researchers with a master’s degree or above who have at least one peer-reviewed publication in field of SS (whether TCM or western medicine or integrative medicine).

##### Process

Firstly, the results from each round of the Delphi survey will be reviewed to decide if they meet the consensus criteria for inclusion or exclusion. Secondly, participants will discuss the outcomes for which there was a lack of agreement. Thirdly, participants will subsequently vote for each lack of agreement outcome for inclusion and exclusion using a format similar to that of the Delphi survey; this will be done anonymously

## Discussion

We summarized the actions of COS into three points: (1) direct action—standardized clinical outcome selection, measurements, and reporting can reduce research bias; (2) indirect action—ensure that outcomes are similar across clinical studies on the same topic, and facilitate the synthesis of secondary evidence, such as meta-analysis; (3) final action—reduce research waste, promote clinical research and clinical practice, ensure patients’ clinical benefits, and reduce family and social burdens.

TCM has many advantages in some respects; however, much of the obtained research outcomes are not accepted by evidence user, which relates to no standardized outcome reporting. Therefore, our study is very necessary. After the final COSs is completed, we will publish this research in a journal to promote communication.

## Supplementary Information

**Additional file 1.** The search strategy example of PubMed.

## Data Availability

Data sharing is not applicable to this article as no datasets were generated or analyzed during the current study.
