# Supplementary material for: Sjogren’s syndrome in clinical trials of traditional Chinese medicine: protocol for the development of a core outcome set
Source: Trials. 2021 Mar 26;22:233. doi: 10.1186/s13063-021-05187-8 (PMC7999517; doi:10.1186/s13063-021-05187-8)
Supplement: Supplementary file 1 — Additional file 1. The search strategy example of PubMed. [file 13063_2021_5187_MOESM1_ESM.docx]

| Databases | Search strategy |
| --- | --- |
| PubMed | #1 "Sjogren's Syndrome"[Mesh]  #2 “Sjogrens Syndrome”[Title/Abstract] OR “Syndrome, Sjogren's”[Title/Abstract] OR “Sjogren Syndrome”[Title/Abstract] OR “Sicca Syndrome”[Title/Abstract] OR “Syndrome, Sicca”[Title/Abstract]  #3 #1 OR #2  #4 Random*[Title/Abstract] OR blind*[Title/Abstract] OR control*[Title/Abstract] OR observation study[Title/Abstract] OR observation studies[Title/Abstract] OR clinical trial*[Title/Abstract]  #5 #3 AND #4 |

Additional file 1 The search strategy example of PubMed
